# Ultraviolet radiation protection potentials of Methylene Blue for human skin and coral reef health

**DOI:** 10.1038/s41598-021-89970-2

**Published:** 2021-05-28

**Authors:** Zheng-Mei Xiong, Xiaojing Mao, Mason Trappio, Chanda Arya, Jasmin el Kordi, Kan Cao

**Affiliations:** 1 Department of Cell Biology and Molecular Genetics, University of Maryland, College Park, MD USA; 2 Mblue Labs, Bethesda, MD USA

**Keywords:** Cell biology, Health care

## Abstract

Methylene blue (MB) is a century-old medicine, a laboratory dye, and recently shown as a premier antioxidant that combats ROS-induced cellular aging in human skins. Given MB’s molecular structure and light absorption properties, we hypothesize that MB has the potential to be considered as a sunscreen active for UV radiation protection. In this study, we tested the effects of MB on UVB ray-induced DNA double-strand breaks in primary human keratinocytes. We found that MB treatment reduced DNA damages caused by UVB irradiation and subsequent cell death. Next, we compared MB with Oxybenzone, which is the most commonly used chemical active ingredient in sunscreens but recently proven to be hazardous to aquatic ecosystems, in particular to coral reefs. At the same concentrations, MB showed more effective UVB absorption ability than Oxybenzone and significantly outperformed Oxybenzone in the prevention of UVB-induced DNA damage and the clearance of UVA-induced cellular ROS. Furthermore, unlike Oxybenzone, MB-containing seawater did not affect the growth of the coral species *Xenia umbellata*. Altogether, our study suggests that MB has the potential to be a coral reef-friendly sunscreen active ingredient that can provide broad-spectrum protection against UVA and UVB.

## Introduction

Methylene blue (MB) is a common laboratory dye and widely used medicine. Since its first synthesis in 1876, MB has been reported to have broad applications in clinical treatments of methemoglobinemia, malaria, vasoplegia, septic shock, and Alzheimer’s disease, etc^1–3^. As a diaminophenothiazine, MB has a low redox potential of 11 mV. This antioxidant property allows for efficient cycling between the oxidized form (MB) and the reduced form leucomethylene blue (MBH2), which facilitates electron transport in the mitochondrial inner membrane and reduces mitochondrial superoxide production^4,5^. The use of MB in skincare helps combat ROS-induced cellular aging^6^. We have previously demonstrated that MB outperforms other leading antioxidants (e.g., N-Acetyl-L-Cysteine (NAC), MitoQ, and mitoTEMPO) in neutralizing free radicals and improving skin longevity^6^. MB also shows promising effects in stimulating the production of healthy skin fibers (e.g., Collagen and Elastin), improving skin hydration, and promoting wound healing. Additionally, MB is environmentally friendly and is highly compatible with marine ecosystems. It is commonly used as an aquaculture supplement to treat injured or sick fish. The properties of MB as an oxygen transporter make it useful in the treatment of known cyanide and nitrite poisoning of aquarium fish.

The two types of UV radiation that reach our skin are UVA and UVB waves, which cause different types of skin damage. UVA waves are longer in wavelength (320–400 nm) and penetrate deeper into the skin, generating high levels of ROS in the dermis and cause photoaging. UVB waves are shorter (280–320 nm) in wavelength and carry higher energy—they cause erythema (i.e., sunburn), and DNA damage/mutations within skin cells (e.g., melanocytes, keratinocytes) that may lead to skin cancer.

Oxybenzone (BP-3) is one of the most commonly used chemical blockers in sunscreen products as a chemical blocker—over 80% of chemical sunscreens contain Oxybenzone or its derivatives^7^. However, multiple studies have shown that Oxybenzone is a significant hazard for aquatic ecosystems and expedites the destruction of coral reefs^8,9^, which are essential for aquatic biodiversity and are considered “the rain forest of the sea”. These compounds have been found entering the ocean environment through wastewater effluent or directly from swimmers wearing sunscreens. In particular, a study published recently showed four major toxic effects of Oxybenzone in early, developing coral: increased susceptibility to bleaching, DNA damage, abnormal skeleton growth (via endocrine disruption), and gross deformities of coral nubbins^8^. Thus, searching for nontoxic Oxybenzone alternatives is critical for protecting reefs and the exacerbating effects posed by climate change and bleaching. In May 2018, Hawaii became the first state to ban chemical sunscreens containing Oxybenzone. Since then, Florida, California, the US Virgin Islands, Australia, Mexico Bonaire, Palau, and Aruba have joined the ever-growing list of concerned states and countries.

Given MB’s phenothiazine ring structure, solubility, and antioxidant property^10^, we speculate that MB can be served as a potential Oxybenzone alternative. In this study, we measured the UV absorption properties of MB in comparison with Oxybenzone and tested the effects of MB on DNA damage and cell death induced by UVB in primary human keratinocytes. To determine the effects of MB on UVA-induced photoaging, we compared the cellular ROS and cell proliferation in skin cells treated with MB, Oxybenzone, or as well as two popular antioxidants in skincare, Vitamin A and Vitamin C. Finally, we compared the effects of MB exposure on a soft coral species *Xenia umbellata* with Oxybenzone.

## Results

### MB, but not Oxybenzone (BP-3), mitigates DNA double-strand breaks induced by UVB irradiation in human keratinocytes

**Figure 1:**
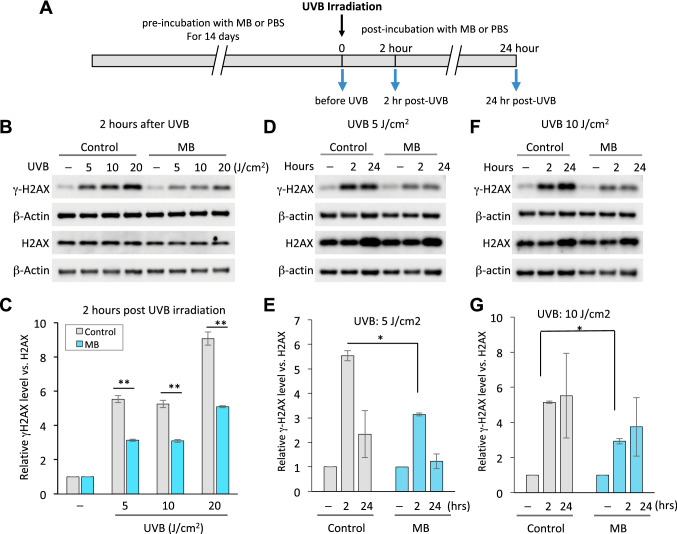
MB alleviates DNA damage induced by UVB irradiation in human keratinocytes. (**A**) Schematic diagram showing the experimental timeline starting from pre-incubation of keratinocytes with 100 nM MB or PBS for 2 weeks, then exposure to UVB light, and ending with cell pellets collection before irradiation (time 0), and 2 or 24 h post-irradiation for Western blotting analysis. (**B**) Representative Western blotting images with anti-γH2AX, H2AX, and β-actin antibodies showing DNA damage response after 2 h of UVB irradiation at different dosages. No UVB control is designated as “⎯”. (**C**) Quantification of γH2AX band intensities shown in (**B**), which is normalized to total H2AX. * *p* < 0.05; ** *p* < 0.01 MB versus Control at the same dose. (**D**,**F**) Representative Western blotting images with anti-γH2AX, H2AX, and β-actin antibodies in human keratinocytes. The cell pellet samples were collected at 0, 2, or 24 h after UVB irradiation at 5 J/cm^2^ (**D**) or 10 J/cm^2^ (**F**). Zero hour control is designated as “⎯”. (**E**, **G**) Quantification of γH2AX band intensities shown in (**D**,**F**), which is normalized to total H2AX. * *p* < 0.05; MB versus Control at the same time point.

Our previous study demonstrated that MB could stimulate the DNA repair process by upregulating DNA damage repair pathway factors, such as PARP1, which potentially can undo the DNA damage caused by UVB radiation^11^. To directly analyze the impact of MB on DNA damage caused by UVB irradiation, we conducted a dosage escalation study. Primary human skin keratinocytes were pre-treated with 100 nM MB for two weeks and then exposed to 0, 5, 10, and 20 s of UVB rays at 1 W/cm^2^, which resulted in irradiation dosages of 0, 5, 10, and 20 J/cm^2^ (Fig. 1A). This range of UVB irradiation intensity was chosen empirically based on the UVB intensities detected on hot summer days at the University of Maryland campus (Supplemental Figure S1A-C).

To quantify the levels of DNA double-strand breaks, we used a well-established DNA damage marker γH2AX^12^. The histone H2AX is phosphorylated to a gamma form (γH2AX) upon DNA double-strand breakage, and the levels of γH2AX positively correlate with the presence of DNA breaks, which is commonly generated by UVB^13^. β-actin was also probed as an internal loading control of each sample, and the total expression of H2AX was measured as well. The DNA damage could be detected as early as two hours post UVB exposure, indicated by elevated γH2AX levels (Fig. 1B). Compared to non-treated controls, skin keratinocytes that were pre-treated with MB showed reduced γH2AX expression upon UVB irradiation at each dosage (Fig. 1C), suggesting that MB provides protection of the skin cells from the UVB damage. Furthermore, to determine the long-term recovery potential in these irradiated cells, we measured γH2AX levels at 2 and 24 h after UVB irradiation (Fig. 1D–G). We found that under a lower dosage (5 J/cm^2^), it was possible for the control cells (PBS pre-treatment) to recover and express the relatively normal level of γH2AX after 24 h (Fig. 1D,E). Under the higher dosage of 10 J/cm^2^ UVB irradiation, the expression of γH2AX continued to increase until at least 24 h after UVB exposure (Fig. 1F,G). In MB pre-treated cells, the magnitude of DNA damage was significantly reduced (Fig. 1D–G).

### MB prevents human keratinocytes from UVB-induced cell death

**Figure 2:**
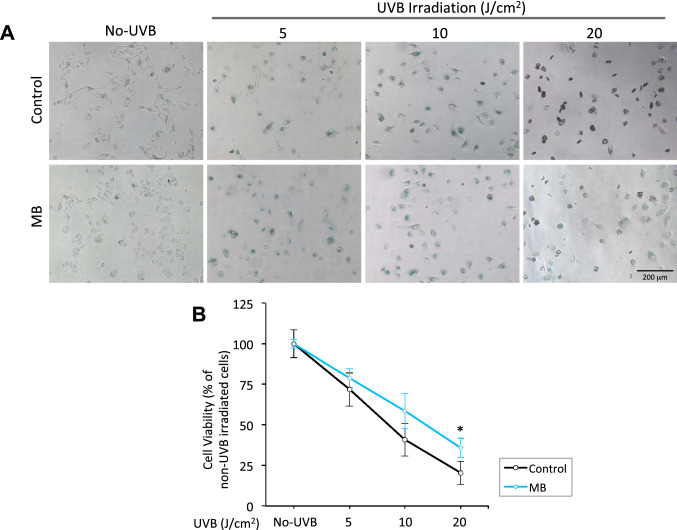
MB prevents human keratinocytes from UVB-induced cell death. (**A**) Representative images of senescence-associated β-galactosidase (SA-β-gal) staining in human keratinocytes 5 days after UVB irradiation at 0, 5, 10, and 20 J/cm^2^. The cells were pretreated with Control (PBS) or 100 nM MB for two weeks before UVB irradiation. Scale bar = 200 μm. (**B**) AlarmaBlue cell viability assay in Control and MB-treated human keratinocytes at day five after UVB exposure at denoted dosages. The cells were pretreated with Control (PBS) or 100 nM MB for two weeks before UVB irradiation. * *p* < 0.05 MB versus Control.

Following the induction of unfixable amounts of DNA damage by UVB, major routes of cell inactivation are either cell senescence or cell death^14^. To further evaluate MB’s ability to protect skin cells from UVB damage, we examined the cell senescence and viability by senescence-associated β-galactosidase (SA-β-Gal) staining and AlamarBlue cell viability assay, respectively. Keratinocytes were first treated with MB or control PBS for two weeks, then subjected to 0, 5, 10, or 20 J/cm^2^ of UVB irradiation and allowed to recover for 5 days. At each UVB dosage, we found that there were an overall healthier appearance and reduced β-Gal staining in the MB-treated cells than in control. A higher percentage of dead keratinocytes were found in the untreated controls, as indicated by the dark staining of the cells (Fig. 2A). Consistently, the MB-treated groups showed significantly higher survival rates after five days of recovery (Fig. 2B).

### MB shows a broad UV absorption capability

**Figure 3:**
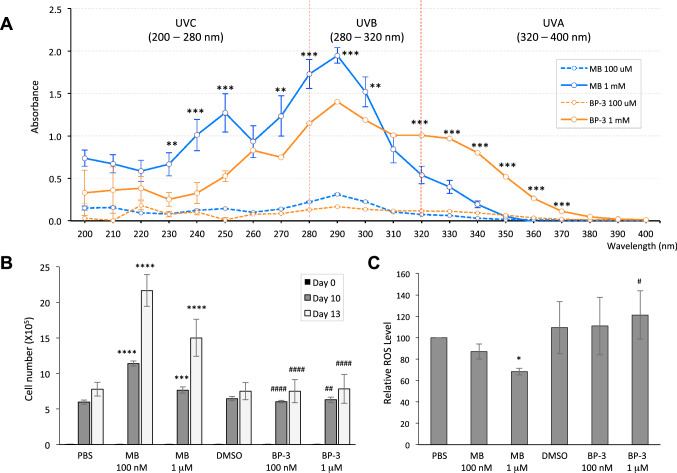
MB is a potent ROS scavenger with stronger UVB absorption than Oxybenzone (BP-3). (**A**) Graphs of UV absorption spectrum of MB and BP-3 at 100 μM and 1 mM. PBS, the solvent of MB, was used to establish blank reading for UV spectrum measurement. DMSO, the solvent of BP-3, at a final concentration of 0.01% in PBS, was measured for adjustment of BP-3 absorbance. (**p < 0.01, *** *p* < 0.001 MB 1 mM vs. BP-3 1 mM). (**B**) Keratinocytes were seeded at the same cell number on day 0 and maintained in culture medium containing PBS, 100 nM or 1 µM MB, 0.01% DMSO (solvent for BP-3), 100 nM or 1 µM BP-3, respectively. Cell counting was conducted on day 10 and day 13 to compare the cell proliferation under different treatments. *** *p* < 0.001; ****p < 0.0001 comparing with PBS on the same day ; ## *p* < 0.01 #### *p* < 0.0001 MB versus BP-3 on the same day and same concentration. (**C**) Keratinocytes pretreated with PBS, 100 nM MB, 1 µM MB, 0.01% DMSO, 100 nM BP-3 and 1 µM BP-3 for 2 weeks were examined for mitochondrial specific superoxide (MitoSOX) levels by FACS on day 14. * *p* < 0.05 comparing with PBS; # *p* < 0.05 MB 1 µM versus BP-3 1 µM.

One of the major characteristics of a chemical sunscreen blocker is its ability to absorb UV rays and convert the rays into heat. Thus, we next measured the absorption characteristics of MB using spectrophotometric analysis and compared the results with BP-3 at the same concentrations of 100uM and 1 mM (Fig. 3A). The result indicated that MB has a broad-spectrum absorption range across UVA, UVB, and UVC wavelengths. It is a more effective UV blocker than BP-3 at the high energy, short wavelength UVB/C range (< 310 nm). For the longer wavelength lights (> 310 nm, UVA), BP-3 absorbs better than MB.

### MB is a more robust ROS scavenger than BP-3, Vitamin A, and Vitamin C in skin cells

UVA rays are longer in wavelength and penetrate deeper into the skin, generating high levels of ROS, leading to skin cell aging and death. To compare the MB’s and BP-3’s effects on combating ROS, next, we conducted the cell proliferation and ROS assays in keratinocytes after two-week treatment with either MB or BP-3 at the 100 nM and 1 µM concentrations (Fig. 3B,C). We observed an increase in cell proliferation in MB-treated human primary keratinocytes, indicating MB is beneficial to overall skin cell longevity. Although BP-3 has an EWG hazard score of 8 (toxic) on the human body, the proliferation study did not reveal any significant inhibitory effects of BP-3 (Fig. 3B), which suggests that at the 100 nM and 1 µM concentrations, BP-3 is probably safe to human skin cells. Moreover, consistent with our previous study^6^, an evident dosage-dependent reduction in ROS levels in MB-treated cells was found (Fig. 3C). In contrast, no significant changes in ROS levels were observed in the BP-3 treated cells compared to Dimethyl Sulfoxide (DMSO)-treated controls, indicating BP-3 is not a ROS scavenger.

**Figure 4:**
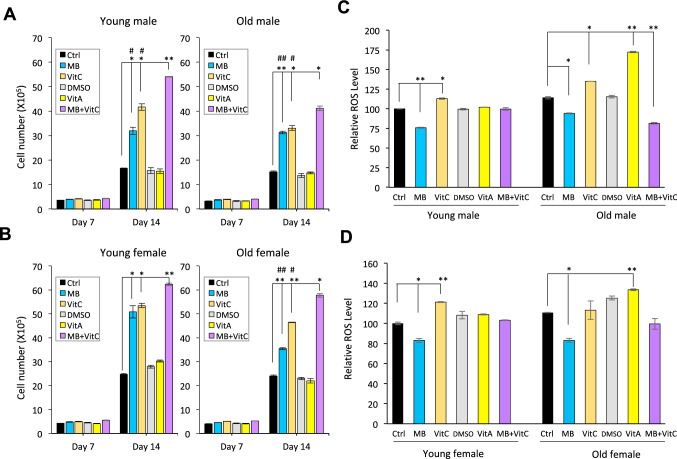
MB is a more effective ROS scavenger than Vitamin A and Vitamin C in human skin fibroblasts. (**A**) Young (Left) and old (Right) male fibroblasts proliferation during the fourteen-day treatment with control PBS, 0.01% DMSO, 100 nM MB, 100 nM Vitamin A, 100 μM Vitamin C, and 100 nM MB + 100 μM Vitamin C, respectively. *p* < 0.05, **p < 0.01 comparing with Control PBS on the same day; # *p* < 0.05 or ## *p* < 0.01 comparing with MB + Vitamin C-treated cells on the same day. (**B**) Young (Left) and old (Right) female fibroblasts proliferation during the fourteen-day treatment with control PBS, 0.01% DMSO, 100 nM MB, 100 nM Vitamin A, 100 μM Vitamin C, and 100 nM MB + 100 μM Vitamin C, respectively. *p* < 0.05, **p < 0.01 comparing with Control PBS on the same day; # *p* < 0.05 or ## *p* < 0.01 comparing with MB + Vitamin C-treated cells on the same day. (**C**) Comparison of the mitochondrial specific superoxide (MitoSOX) levels in young and old male fibroblasts after the treatment of control PBS, 0.01% DMSO, 100 nM MB, 100 nM Vitamin A, 100 μM Vitamin C, or 100 nM MB + 100 μM Vitamin C for 4 weeks. *p* < 0.05, **p < 0.01 comparing with Control PBS. (**D**) Comparison of the final mitochondrial specific superoxide (MitoSOX) levels in young and old female fibroblasts after the treatment of PBS control, 0.01% DMSO, 100 nM MB, 100 nM Vitamin A, 100 μM Vitamin C, and 100 nM MB + 100 μM Vitamin C for 4 weeks. *p < 0.05, **p < 0.01 comparing with Control PBS.

To further investigate MB’s capability as a ROS scavenger and UVA protective agent for the skin, we obtained four human skin fibroblast cell lines from young male, old male, young female, and old female donors (Detailed cell line information in Supplemental Table S1). We evaluated the effectiveness of MB in comparison with Vitamin A and Vitamin C, both of which are popular antioxidants in skincare products^15^. We first monitored the cell proliferation for a 2-week time frame. In this experiment, we observed that MB enhanced the cell proliferation greatly in all four lines with comparable effectiveness to Vitamin C (Fig. 4A,B). Vitamin A, conversely, did not affect the cell proliferation rates (Fig. , B). The combination of MB and Vitamin C synergistically boosted the cell proliferation capacities even higher in all four lines (Fig. 4A,B). Next, we measured the final mitochondrial ROS after a four-week treatment period. Surprisingly, MB was the only treatment that consistently reduced the mitochondrial ROS in all four cell lines. The combination of MB and Vitamin C worked more effectively in old fibroblasts in ROS clearance, while in young cell lines, this combination has less of an effect (Fig. 4C,D).

### MB is non-toxic to coral health as compared to Oxybenzone (BP-3)

**Figure 5:**
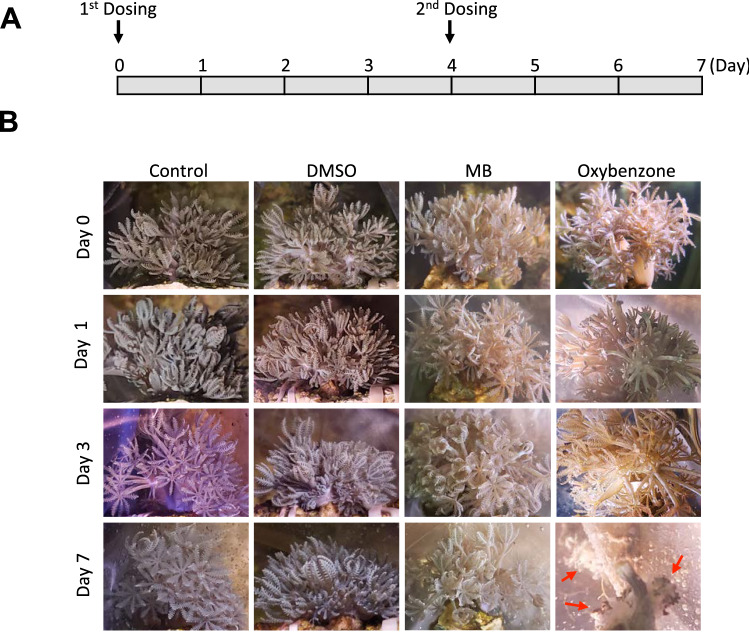
MB is non-toxic to coral health compared to oxybenzone (BP-3). (**A**) Schematic experimental timeline showing the two administrations of PBS control, DMSO 0.005%, 1 μM MB, and 1 μM Oxybenzone on day 1 and day 4, respectively. (**B**) Representative images of coral colonies growing on the stone base in the seawater containing different chemicals on day 0 (before adding chemicals), day 1, day 3, and day 7. Red arrows indicate the death of corals in the Oxybenzone-treated experiment on day 7.

The results of the previous experiments support that MB’s potential as an effective protectant for both UVA and UVB. To determine MB’s effects on coral reefs, we grew the soft coral species *Xenia umbellata* in seawater containing either 1 μM MB or 1 μM Oxybenzone for one week (Fig. 5A). We observed that by Day 7 in the Oxybenzone -treated coral, tissue debris was found at the bottom of the coral incubating bag (Fig. 5B). Also, following the collection of the tissue debris, we observed the lack of pulsing from the treated coral. Pulsing in the *Xenia* coral completely ceased (Supplementary videos). The coral colony appeared limp, disintegrated, and fell away from the light above (Fig. 5B), possibly indicating an expulsion of symbiotic zooxanthellae species from within the coral to the treated seawater^16^. None of these observations were found within the MB-treated corals (Supplementary videos).

## Discussion

The two types of sunscreens on the market include physical blockers that scatter UV rays, i.e., Zinc Oxide, Titanium Dioxide, and chemical absorbers, such as Oxybenzone, that absorb UVA and UVB rays; both are designed to prevent UV rays from penetrating the skin. Although this traditional method for sun protection is mostly effective, even the sunscreens with the highest SPFs can only stop up to 98% of UVB rays from penetrating the skin^17^. The remaining UV waves that penetrate the skin can still cause damage. Moreover, penetration of UV waves into the skin can have positive health benefits, such as the generation of Vitamin D. Thus, we argue that new sun protection products should also be geared towards reversing the negative effects of UV radiations in addition to blocking UV waves.

MB is the first man-made compound that was synthesized in 1876^18^. It has been approved for medical use by the FDA, and it is also on the WHO list of safe and essential medicines. Our previous work indicated that MB activates the DNA damage repair pathway inside the cell, possibly through activating DNA damage repair pathways^11^. In this study, we tested the ability of MB on mitigating UVB -induced DNA damages in primary human keratinocytes. We found that MB treatment reduced the overall levels of DNA double strand breaks caused by UVB irradiation and increased cell survival rate (Figs. 1, 2). Absorption spectroscopic analysis revealed that MB has a broad-spectrum light absorption range covering from UVA, UVB to UVC wavelength (Fig. 3A). Thus, MB has the desired abilities of both UVB blocking and DNA damage mitigation. It not only absorbs UVB but also reduces the DNA damages induced by UVB, thereby promoting cell survival post UVB irradiation.

UVA radiation significantly increases levels of ROS inside the skin. These high levels of ROS result in damage to skin cells and their surrounding connective tissue, which leads to the physical signs (e.g., wrinkles, reductions in skin elasticity) of premature photoaging. Endogenous antioxidants within the skin are depleted during elevated levels of UVA generated ROS. As a result, many anti-aging skincare creams deliver exogenous antioxidants to help combat ROS. Vitamin A (commonly known as Retinol in skincare) and Vitamin C are popular active antioxidants used in skincare. In the comparative studies demonstrated in Figs. 3B, C and 4, both MB and Vitamin C promoted cell proliferation, but BP-3 and Vitamin A had little effects at the tested concentrations (Figs. 3B and 4A,B). Among these four compounds, MB is no doubt the most effective ROS scavenger in human skin cells (Figs. 3C and 4C,D). Additionally, we found that when MB is combined with Vitamin C, a positive synergistic benefit was observed. The MB plus Vitamin C treated cells showed the highest skin cell proliferation rate and lowest cellular stress as determined by mitochondrial ROS assay. Furthermore, this synergistic effect on combating ROS is more prominent in old skin cells than in young cells (Fig. 4C,D). These results indicate the combination of MB and Vitamin C could be used in skincare to deliver optimal anti-aging effects.

Besides MB’s UVA and UVB blocking and mitigating effects, the soft coral experiment indicated that at a high concentration (1uM), MB posed no negative effects on coral health and growth (Fig. 5), which was not a surprise as MB has been commonly used by fish hobbyists as an aquaculture supplement. In contrast, Oxybenzone, which was reported to pollute the marine ecosystem previously^8,9^, impaired the growth of coral reefs and caused coral bleaching and death in our experiments.

Altogether, these results suggest that MB has great potential to serve as a coral reef-friendly, Oxybenzone alternative in sunscreen products. It will bring many benefits that outweigh most of the current sunscreen actives, particularly relative to Oxybenzone. Firstly, as a potent antioxidant, the MB-based sunscreen may protect skin from ROS and photoaging caused by UVA rays^6^, which is lacking in current sunscreen products^19^. Secondly, MB has a broad-spectrum absorption covering UVA and UVB and is able to lessen the UVB-induced DNA damages and protect cells from cell death, possibly by activating DNA repair pathways. Therefore, MB has the abilities of both UVB blocking and UVA/B damage mitigation. Lastly and most importantly, the MB-based sunscreen is safe for marine life and ecosystems, especially for the already endangered coral reefs. In the US, the FDA regulates sunscreens as an over-the-counter (OTC) and has established a monograph for active ingredients. New ingredients have to undergo rigorous and costly safety testing to be considered for inclusion in the sunscreen monograph. Future work will include conducting the required FDA studies in order to submit an application for full inclusion of Methylene Blue in the sunscreen monograph.

## Methods

### Cell culture and drug treatment

The normal human primary epidermal keratinocyte line (PCS-200-010) was obtained from ATCC. Keratinocytes were thawed and cultured in Dermal Cell Basal Medium (PCS200030, ATCC) supplemented with Keratinocyte Growth Kit (PCS200040, ATCC) at 37 °C with 5% CO_2_. Four of normal human skin fibroblasts were purchased from Coriell Institute (detailed information described in Table S1). All fibroblast cells were cultured in MEM (Life Sciences) supplemented with 15% FBS (Gemini Bio-Products) and 2 mM L-glutamine (Life Sciences) at 37 °C with 5% CO_2_.

Methylene blue (1%, Akron) and L-Ascorbic acid (Vitamin C, A4544, Sigma-Aldrich) were prepared in PBS. Retinoid acid (Vitamin A, R2500, Sigma-Aldrich), Oxybenzone (BP-3, H36206, Sigma-Aldrich), and Octinoxate (PHR1080, Sigma-Aldrich) were dissolved in DMSO (final concentration 0.01% in culture medium or 0.005% in seawater).

### Drug treatment and UVB irradiation

Keratinocytes were seeded in culture dishes at a density of 2500 cell/cm^2^ and fed with fresh medium containing MB or vehicle PBS on next morning to start the treatment for 1 or 2 weeks before UVB irradiation. The cells were subcultured every 2 or 3 days when the culture has reached about 80% confluency.

UVB treatment on human keratinocytes was conducted in a tissue culture hood with a handheld UV lamp emitting a peak wavelength of 302 nm (UVM-57, Analytik Jena). The UVB intensity was measured each time before irradiation with a digital Radiometer (UVP UVX™, Analytik Jena) that is attached to a radiometer sensor (UVX-31 300NM, Analytik Jena). Briefly, the cells were gently rinsed out once with PBS then exposed to UVB at the intensity of 1 W/cm^2^ for 0 s, 5 s, or 10 s, which is equal to various doses at 0, 5, or 10 J/cm^2^, respectively. The cells were then immediately fed with fresh medium containing MB or PBS and collected after 2- or 24-h for western blotting analysis.

### Western blotting

Western blotting was done following the same protocol as described in Xiong et al.^14^ Antibodies used in this study included: γH2AX (Ab11174, Abcam), β-actin (A3854, Sigma-Aldrich), H2AX (600201, Biolegend).

### Senescence associated β-Galactosidase activity assay

We followed the manufacturer’s protocol (#9860; Cell Signaling). Briefly, keratinocytes cells grown on a six-well plate were fixed in 1X fixative solution for 10 min and then stained overnight at 37 °C with the β-galactosidase staining solution at pH 6.0 for 15 h. Bright-field and phase contrast images were taken with a Zeiss AX10 microscope and a SPOT PURSUIT camera.

### AlamarBlue cell viability assay

Cells growing in a culture plate were incubated in complete medium containing 10% alamarBlue reagent (DAL1025, ThermoFisher Scientific) for 3 h and the plates were read by the fluorescence spectrophotometer using 570/590 nm (excitation/emission) filter settings (Molecular Devices Spectramax M5).

### UV spectra measurement

The absorbance of MB or BP-3 to broad UV–VIS spectrum was measured in triplicate using NanoDrop 2000 spectrophotometer (ThermoFisher Scientific). Briefly, 2 μL of MB or BP-3 was pipetted onto the pedestal for measurement after the establishment of a blank using PBS under the setting of UV–Vis application. The solvent of BP-3, DMSO, at a final concentration of 0.01% in PBS, was also measured for adjustment of BP-3 absorbance.

### Flow cytometry analysis for mitochondrial ROS

Mitochondrial superoxide was measured following the same protocol as described in Xiong et al.^14^ Briefly, cells cultured on 60-mm dishes were incubated with fresh complete medium containing 5 μM MitoSOX Red (Life Technologies, M36008) at 37 °C. After 30 min, stained cells were harvested by trypsin digestion and rinsed twice with PBS. Single cell suspensions in 400 ml PBS were prepared for FACS analysis (FACS Canto II; BD). MitoSOX Red was excited by a laser at 488 nm, and the data was collected at 582 ± 21 nm.

### Coral growth and treatment

Soft Corals (*Xenia umbellata*) frags were purchased from ORA. The corals were cultivated in a 12 gallon NanoCube aquarium with a 12 h light cycle (JBJ). Reef Salts (Instant Ocean) were used to bring the specific gravity of the aquarium and treatment water to 1.024. Corals were fed with liquid and dry feeds (Polyp Lab) and supplemented with potassium iodide (SeaChem). Water temperature was held constant at 25.5 °C by a submersible HT10 thermometer (Tetra). Chemical tests on coral health started after one month acclimation period. On day 0, each coral colony was gently transferred into a 2 mil thick resealable bag (VWR) containing 600 mL seawater. The bag was then magnetically mounted on the wall of the aquarium (Siam). The coral colony was imaged two hours after settlement and various chemicals were administered directly to the bag water on day 0 and day 4.

### Statistical analysis

Results are presented as the mean ± standard deviation. Data statistical analysis was performed using one-way ANOVA with GraphPad Prism 9, and a *p* value less than 0.05 was considered significant.

## Supplementary Information

Supplementary Legends.

Supplementary Information.

Supplementary Video 1.

Supplementary Video 2.
