# Supplementary material for: Ultraviolet radiation protection potentials of Methylene Blue for human skin and coral reef health
Source: Sci Rep. 2021 May 28;11:10871. doi: 10.1038/s41598-021-89970-2 (PMC8163870; doi:10.1038/s41598-021-89970-2)
Supplement: Supplementary file 1 — Supplementary Legends. [file 41598_2021_89970_MOESM1_ESM.docx]

**Supplemental Data**

Ultraviolet radiation protection potentials of Methylene Blue for human skin and coral reef health

Zheng-Mei Xiong*^, 1^, Xiaojing Mao*^, 1^, Mason Trappio ^1^, Chanda Arya ^1,2^, Jasmin el Kordi ^2^, and Kan Cao ^1,2, #^

^1^ Department of Cell Biology and Molecular Genetics, University of Maryland, College Park, MD, USA

^2^ Mblue Labs, Bethesda, MD, USA

*: Equal contributors

# Corresponding Author:

Kan Cao, Ph.D.

Email: [kcao@umd.edu](mailto:kcao@umd.edu)

Phone: 301-405-3016

Supplemental Figure legends

Figure S1. Measurement of UVB intensity in Hornbake Plaza on the University of Maryland College Park campus

(A) The UVB intensity from the sun was measured at four time points (9:00 am, 12:00 pm, 3:00 pm and 6:00 pm) in the exact same spot of Hornbake Plaza on 27^th^ to 29^th^, August 2018. * The sun was hidden behind a thick cloud. Red dashed line indicates the chosen experimental setting. The local temperature (B) and weather (C) at different time points were recorded accordingly while UVB was measured.

Figure S2: Original gel images of all western blots shown in figures.

Supplemental Table 1. Information for Human Fibroblast Cell Lines

Supplemental Videos. Movies of live coral colonies growing in MB- or Oxybenzone-containing seawater on day 7
