# Supplementary material for: Ultraviolet radiation protection potentials of Methylene Blue for human skin and coral reef health
Source: Sci Rep. 2021 May 28;11:10871. doi: 10.1038/s41598-021-89970-2 (PMC8163870; doi:10.1038/s41598-021-89970-2)
Supplement: Supplementary file 2 — Supplementary Information. [file 41598_2021_89970_MOESM2_ESM.pdf]

Fig S1: UVB measurement in Hornbake Plaza at UMD, College Park Campus

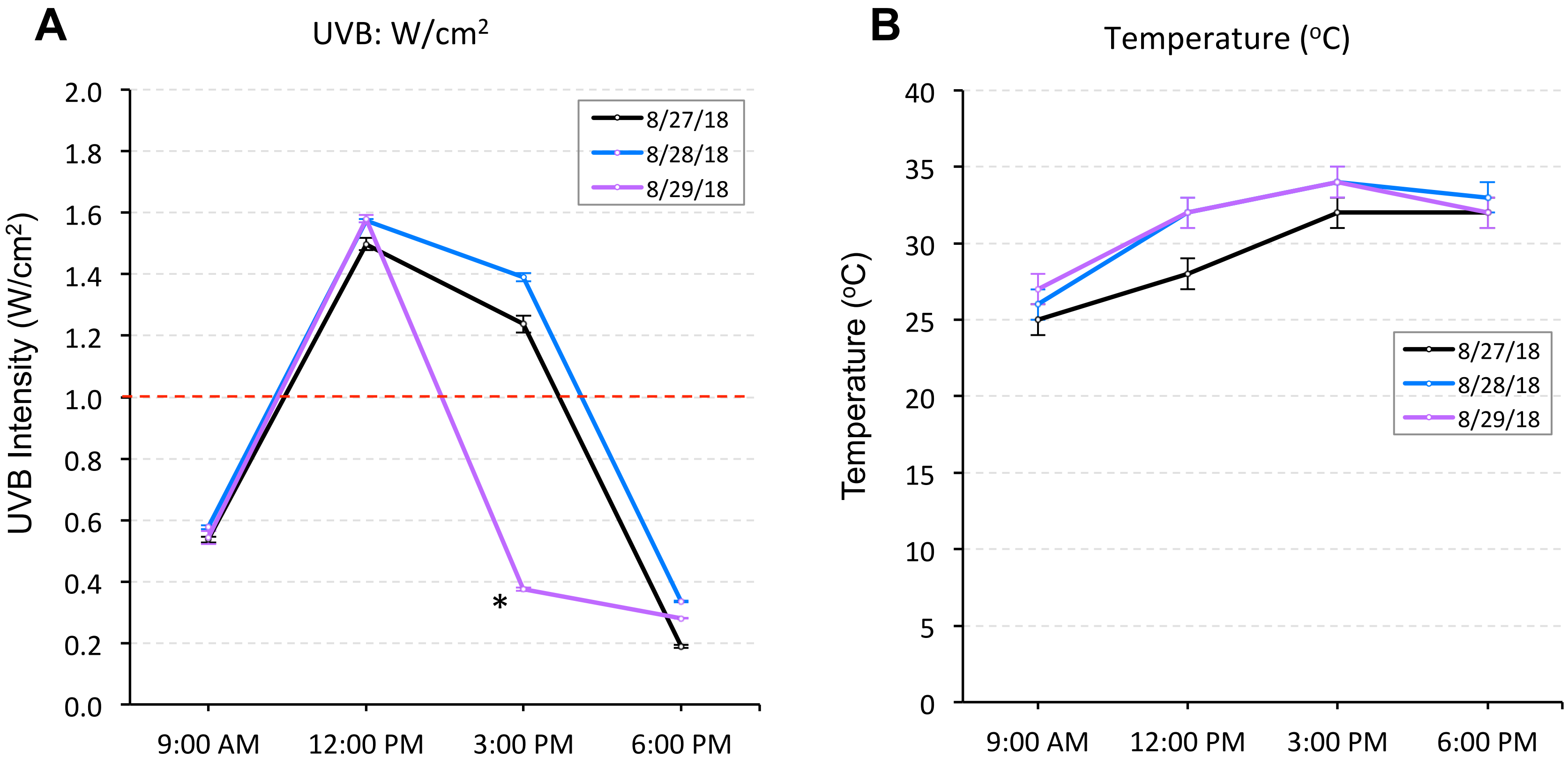

C

Weather	9:00 AM	12:00 PM	3:00 PM	6:00 PM
8/27/18	Sunny	Partly Cloudy	Partly Cloudy	Partly Cloudy
8/28/18	Sunny	Sunny	Partly Cloudy	Partly Cloudy
8/29/18	Partly Cloudy	Sunny	Partly Cloudy	Sunny

Figure S2: Original Western Images shown in Figure 1A

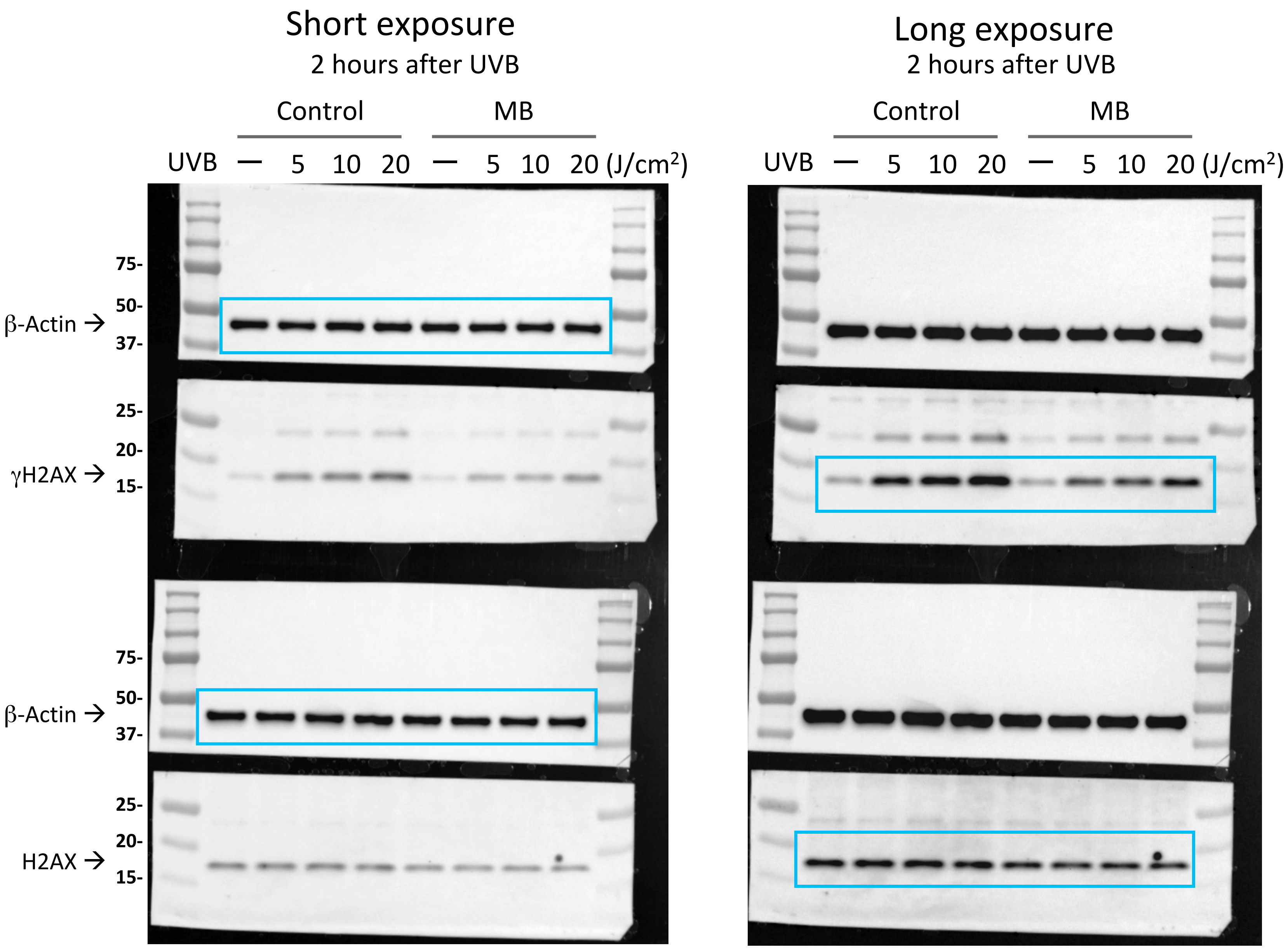

The cropped band images in blue boxes were presented Figure 1A.

Original Western Images

Figure 1C
UVB 5 J/cm²

Figure 1E
UVB 10 J/cm²

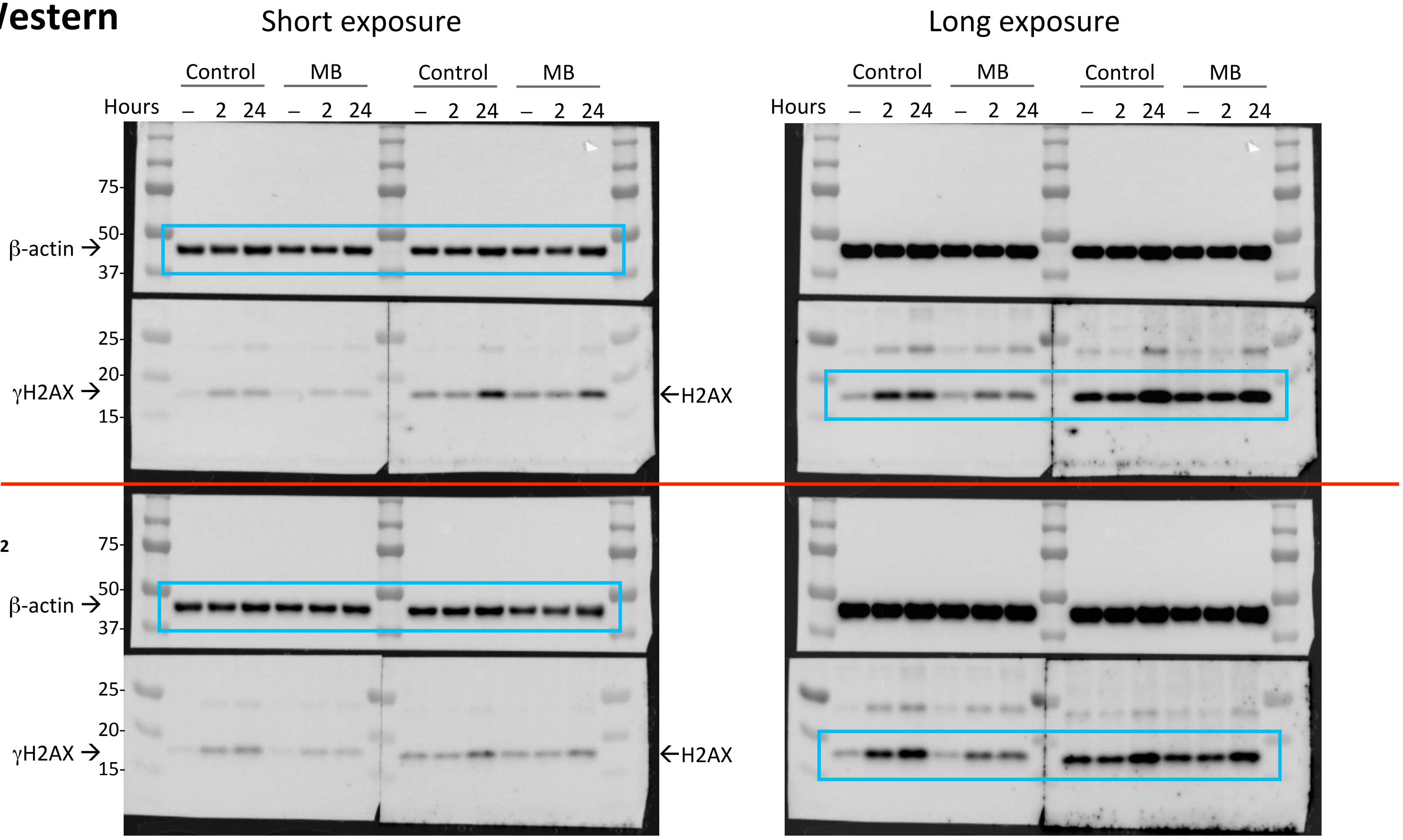

Supplemental Table 1. Information for Human Fibroblast Cell Lines

Cell line (Name in paper)	Passage Number (Starting → Ending)	Gender	Donor Age	Race	Resource
AG09266 (1-YM)	P13 → P19	Male	26 yrs	Caucasian	Coriell*
AG11695 (2-OM)	P13 → P19	Male	82 yrs	Caucasian	Coriell
AG08434 (3-YF)	P13 → P19	Female	29 yrs	Caucasian	Coriell
AG11725 (4-OF)	P13 → P18	Female	84 yrs	Caucasian	Coriell

1-YM: 1-Young Male 3-YF: 3-Young Female
2-OM: 2-Old Male 4-OF: 4-Old Female

*Coriell Institute for Medical Research
